# Neuroimaging in Frontotemporal Dementia: Heterogeneity and Relationships with Underlying Neuropathology

**DOI:** 10.1007/s13311-021-01101-x

**Published:** 2021-08-13

**Authors:** Bradley T. Peet, Salvatore Spina, Nidhi Mundada, Renaud La Joie

**Affiliations:** Memory and Aging Center, Department of Neurology, Weill Institute for Neurosciences, University of California, San Francisco, CA USA

**Keywords:** Frontotemporal dementia, Frontotemporal lobar degeneration, Behavioral variant frontotemporal dementia, Semantic variant primary progressive aphasia, Semantic dementia, Nonfluent agrammatic variant primary progressive aphasia, Progressive nonfluent aphasia, Tau, FTLD-tau, TDP-43, FTLD-TDP, Neuroimaging, Magnetic resonance imaging, Positron emission tomography

## Abstract

Frontotemporal dementia encompasses a group of clinical syndromes defined pathologically by degeneration of the frontal and temporal lobes. Historically, these syndromes have been challenging to diagnose, with an average of about three years between the time of symptom onset and the initial evaluation and diagnosis. Research in the field of neuroimaging has revealed numerous biomarkers of the various frontotemporal dementia syndromes, which has provided clinicians with a method of narrowing the differential diagnosis and improving diagnostic accuracy. As such, neuroimaging is considered a core investigative tool in the evaluation of neurodegenerative disorders. Furthermore, patterns of neurodegeneration correlate with the underlying neuropathological substrates of the frontotemporal dementia syndromes, which can aid clinicians in determining the underlying etiology and improve prognostication. This review explores the advancements in neuroimaging and discusses the phenotypic and pathologic features of behavioral variant frontotemporal dementia, semantic variant primary progressive aphasia, and nonfluent variant primary progressive aphasia, as seen on structural magnetic resonance imaging and positron emission tomography.

**Supplementary Information:**

The online version contains supplementary material available at 10.1007/s13311-021-01101-x.

## Introduction

Frontotemporal dementia (FTD) is a group of clinically and pathologically heterogeneous syndromes that, historically, have been challenging to diagnose. This challenge is partly due to the rarity of these conditions and lack of familiarity by physicians, however, diagnostic accuracy is also complicated by the complexity and wide range of clinical manifestations of the various FTD syndromes. The ability to visualize the brain in vivo has been fundamental to understanding the pathophysiology of FTD and other neurodegenerative diseases. Historically, neuroimaging was used to “rule out” other potential pathologies responsible for or contribute to cognitive impairment. However, our understanding of neurodegenerative diseases and the development of new neuroimaging techniques and more sophisticated analysis methods have expanded dramatically over the past decades, ultimately leading to the identification of neuroimaging signatures associated with specific neurodegenerative diseases. These advancements have led to neuroimaging being considered a core clinical investigative tool in evaluating cognitive disorders [1–6].

## Frontotemporal Dementia—Core Clinical Syndromes

Frontotemporal dementia is a spectrum of syndromes characterized by progressive deficits in behavior, language, and cognition associated pathologically with frontotemporal lobar degeneration (FTLD). Clinically, FTD is divided into three prototypical clinical syndromes based on the features most prominent early in the disease course. These three syndromes are the behavioral variant of FTD (bvFTD), the semantic variant of primary progressive aphasia (svPPA), and the nonfluent/agrammatic variant of PPA (nfvPPA).

Additionally, several other clinical syndromes with overlapping features with the core FTD symptomatology are included within the FTD clinical spectrum at large, and include FTD with motor neuron disease (FTD-MND), progressive supranuclear palsy syndrome (PSP-S), and corticobasal syndrome (CBS) [7–9]. In the case of PSP and CBS, the terminology can be confusing because of the unperfect clinical-neuropathological correspondence. An important distinction must be made between the clincal *syndrome* and the underlying *neuropathology*, as PSP-S and CBS can result from various neuropathological entities. For example, CBS is a syndrome that can result from corticobasal *degeneration* (CBD), Alzheimer’s disease, or *GRN* mutations among others, whereas CBD neuropathology can manifest as a CBS, PSP-S, bvFTD, or nfvPPA syndrome. The focus of this review will be primarily on the three prototypical clinical syndromes, bvFTD, svPPA, and nfvPPA, and the underlying pathology associated with these syndromes.

### Behavioral Variant Frontotemporal Dementia

Behavioral variant FTD is the most common form of FTD and makes up more than 50% of all FTD cases [10]. It is characterized by early deficits in behavior and executive functioning [4, 11]. The mean age of onset is 58 years with a standard deviation of 11 years [4]. The incidence is 2.7–4.1 per 100,000 person-years, and the prevalence is 15–22 per 100,000 person-years [12]. The time between symptom onset and the initial evaluation is slightly over 3 years, with a standard deviation of about 3 years [4]. The duration of illness is about 8 years from the time of symptom onset, with a standard deviation of 4 years [4, 13].

The behavioral changes typically seen in bvFTD, including disinhibition, apathy, lack of empathy, perseverative or compulsive behavior, and hyperorality, often become severe as the disease progresses [4, 11]. Behavioral disinhibition occurs early in the disease course in about 76% of cases [4]. Disinhibition is often the most salient feature of bvFTD and can have various manifestations, such as impulsivity, socially inappropriate behavior, or loss of manners or decorum [4, 14, 15]. Apathy and inertia are early features in about 84% of cases [4] Apathy is characterized by a reduction in goal-directed behavior, goal-directed cognitive activity, diminished emotional reactivity, or social engagement [16–18], whereas intertia is characterized by a reduction in initiation of behavior [4]. The initial manifestations of apathy and inertia may be subtle; however, motivation and spontaneity is often increasingly apparent as the disease progresses and, in some cases, can significantly limit the patient’s ability to independently perform basic activities of daily living [16–21]. Perseverative, stereotyped, or compulsive/ritualistic behaviors occur early in the disease course in about 71% of cases [4] and may manifest as simple repetitive movements or vocalizations, such as eye blinking, throat clearing, or tapping, or more complex behaviors such as collecting certain objects, hoarding, repetitive storytelling, or frequent unnecessary trips to the restroom [4, 22–27]. Loss of empathy or sympathy is an early symptom in about 73% of cases [4], and often manifests as a lack of concern for the feelings of others or a reduction in social interaction [4, 28–31]. The indifference exhibited by those with bvFTD can lead to a great deal of emotional stress for the caregiver, often the patient’s spouse, due to difficulty connecting with the patient emotionally [21, 32, 33]. Hyperorality and dietary changes occur early in the disease course in about 59% of cases [4]. A range of disordered eating behaviors have been described in bvFTD, including increased appetite, binge eating, and changes in food preferences, often with a preference for carbohydrates. In severe cases, oral exploration and ingestion of inedible objects can occur [34–36].

### Semantic Variant Primary Progressive Aphasia

The semantic variant of PPA is one of two FTD language variants and is characterized by a progressive loss of semantic knowledge of words [37]. The age of onset is typically between 55 and 70 years [38]. Information regarding the incidence and prevalence of svPPA is limited, though the available data indicates a prevalence of about 4–8 per 100,000 [38]. The svPPA entity is very close to the previously developed “semantic dementia” diagnostic category [11, 39], and most patients will fulfill both sets of criteria; see Mesulam et al. [40] for more discussion on the nuances between these concepts.

Individuals with svPPA have difficulty recalling the meaning of words, which worsens as the disease progresses over time [4]. Additionally, they often experience severe anomia, impaired comprehension of single words, and surface dyslexia and dysgraphia [4]. Notably, repetition, grammar, and motor speech are typically spared [41].

### Nonfluent Variant Primary Progressive Aphasia

The nonfluent/agrammatic variant of PPA is characterized by agrammatic or effortful, halting speech with inconsistent speech sound errors and distortions [41]. The mean age of onset is about 60 years, though the age of onset is broad, ranging from about 40 to 80 years [10]. Like svPPA, incidence and prevalence data are lacking, though extrapolation of FTLD autopsy data suggests an incidence of 0.4–0.7 per 100,000 and a prevalence of 0.5–3 per 100,000 [42].

Agrammatism secondary to difficulty processing the grammatical components of speech is a core feature of nfvPPA and is the most significant contributor to nonfluent speech [43]. Other factors that impact fluency include deficits in executive functioning and impaired motor-speech planning [44, 45]. Impaired comprehension of syntactically complex sentences is also commonly observed. In contrast to svPPA, single-word comprehension and object knowledge are typically spared [41, 44].

Apraxia of speech is another core feature of nfvPPA and is characterized by deficits in motor speech planning. Difficulty with motor planning of speech impacts an individual’s ability to coordinate complex oral movements and leads to phonetic or prosodic speech abnormalities [46, 47]. It is important to note that apraxia of speech may occur as a manifestation of nfvPPA [45, 46], but may also occur in the absence of any other symptoms [46]. If apraxia of speech occurs as the sole manifestation of a neurodegenerative process, this is termed primary progressive apraxia of speech (PPAOS) [46]. PPAOS can be divided into three subtypes, reflecting the dominant clinical characteristics of affected individuals. Sound substitutions and additions characterize the phonetic subtype, and typically worsen with increased utterance length, the number of syllables, or word complexity. The prosodic subtype is characterized by syllable segmentation within multisyllabic words or across words in phrases and lengthened intersegment durations between syllables, words, or phrases [46–48]. Furthermore, nfvPPA without apraxia of speech appears to be a distinct clinical syndrome [49].

## FTLD Neuropathology and Genetics

Frontotemporal lobar degeneration is a neuropathological entity characterized by neuronal loss, gliosis, and progressive neurodegeneration predominantly affecting the frontal and temporal lobes [50–52]. The pathologic protein aggregates that form in FTLD are primarily associated with three major molecular classes: TAR-DNA-binding protein 43 (FTLD-TDP), tau (FTLD-tau), and the group of fused in sarcoma protein (FUS), Ewing’s sarcoma protein (EWS), and TATA-binding protein-associated factor 15 protein (TAF15) (collectively known as FTLD-FET). These molecular classes can be further divided into specific molecular subtypes. Molecular subtypes are generally associated with more than one clinical phenotype, which presents a significant challenge when attempting to classify FTLD neuropathology with the various FTD clinical syndromes (Table 1). Furthermore, Alzheimer’s disease accounts for a small percentage of cases in each of the core FTD syndromes. The complexity between the clinical syndromes of FTD and the underlying neuropathology is demonstrated in a 2017 study by Perry et al. [53], which examined the clinical, pathologic, and neuroimaging findings in cases of bvFTD. This study showed numerous pathologic substrates, including Pick’s disease, corticobasal degeneration (CBD), PSP, FTLD-TDP type A, B, C, and D, FTLD-FUS, and Alzheimer’s disease, could result in the bvFTD clinical syndrome. Furthermore, each pathological subtype was found to be associated with partially distinct patterns of atrophy and clinical symptoms.

**Table 1:** Frontotemporal dementia core clinical phenotypes

FTD variant	Clinical features	Neuroanatomy	Most frequent FTLD pathology subtypes	Common genetic mutations
bvFTD	Behavioral disinhibition; apathy; loss of empathy; compulsive behavior; dietary changes; executive dysfunction	Bilateral frontal lobes and anterior temporal lobes	FTLD-TDP type A, B, C FTLD-tau (PiD, CBD, PSP, GGT, *MAPT*) FTLD-FUS	*C9ORF72*, *GRN*, *MAPT*, *VCP*
svPPA	Impaired confrontation naming; impaired single-word comprehension; impaired object knowledge; surface dyslexia/dysgraphia	Bilateral anterior temporal lobes, typically left > right	FTLD-TDP type C FTLD-Tau (PiD, GGT)	–
nfvPPA	Agrammatism; effortful, halting speech with speech sound errors; impaired comprehension of syntactically complex sentences	Left inferior frontal gyrus and insula	FTLD-TDP type A FTLD-Tau (PiD, CBD, PSP)	*GRN*

*bvFTD* behavioral variant frontotemporal dementia, *CBD* corticobasal degeneration, *FTLD* frontotemporal lobar degeneration, *GGT* globular glial tauopathy, *nfvPPA* nonfluent/agrammatic variant primary progressive aphasia, *PiD* Pick’s disease, *PSP* progressive supranuclear palsy, *svPPA* semantic variant primary progressive aphasia

### FTLD-TDP

The TAR-DNA-binding protein of 43 kDa (TDP-43) is a protein encoded by the *TARDBP* gene located on chromosome 1p36.2. TDP-43 is involved in many cellular processes, including RNA splicing, stabilization, transport, transcription, and translation [54, 55]. The majority of TDP-43 is found within the nucleus of neurons; however, it shuttles between the nucleus and cytoplasm under normal physiologic conditions, and up to 30% of TDP-43 can be found in the cytoplasm [51, 56, 57]. In FTLD-TDP, TDP-43 is translocated from the nucleus to the cytoplasm, where it aggregates and forms pathologic intracellular inclusions [51, 57]. The cortical laminar distribution and morphology of the inclusions are variable, resulting in the various FTLD subtypes [51, 58, 59]. The FTLD-TDP subtype terminology can be confusing as multiple classification schemes have been developed and used in the literature. In this review, we use the harmonized classification system proposed in 2011 by Mackenzie et al. [59]. In studies published before 2011, earlier classification systems proposed by Mackenzie et al. [60] and Sampathu et al. [61] were used.

FTLD-TDP type A is characterized pathologically by the presence of numerous short dystrophic neurites and crescentic or oval neuronal cytoplasmic inclusions concentrated primarily in upper cortical layers II/III, as well as moderate numbers of lentiform neuronal intranuclear inclusions [62–64]. The most common clinical phenotypes include nfvPPA, bvFTD, and rarely FTD-MND [62]. FTLD-TDP type B is characterized by a moderate number of neuronal cytoplasmic inclusions and sparse dystrophic neurites throughout all cortical layers [62–64]. The most common clinical phenotypes include bvFTD and FTD-MND [62]. FTLD-TDP type C is characterized by the accumulation of TDP aggregates within elongated dystrophic neurites and rare neuronal cytoplasmic inclusions [62–64]. FTLD-TDP type C is always associated with anterior temporal lobe degeneration, and therefore the most common clinical phenotypes include svPPA and bvFTD [62]. FTLD-TDP type D is characterized by numerous short dystrophic neurites and frequent lentiform neuronal intranuclear inclusions [62–64]. Type D is always associated with a multisystem proteinopathy caused by mutations in the *valosin-containing protein* (*VCP*) gene, which cause a variable phenotypical expression of inclusion-body myopathy, Paget’s disease of the bone, and FTD [65]. A fifth FTLD-TDP subtype, type E, was described in 2017 by Lee et al. [62] in which a series of seven cases demonstrated granulofilamentous neuronal inclusions and grain-like deposits in all neocortical layers that did not meet the 2011 classification scheme proposed by Mackenzie et al. [59].

### FTLD-Tau

Microtubule-associated protein tau (Tau) is a protein encoded by the *MAPT* gene located on chromosome 17q21 and is involved in the assembly and stabilization of microtubules [66–68]. Six tau isoforms are generated from *MAPT* through alternative mRNA splicing of exons 2, 3, and 10. Exon 10 encodes one of the repeat-containing sequences of the microtubule-binding domain, and alternative splicing gives rise to tau isoforms containing either three (3R) or four (4R) microtubule-binding repeats, each of which group contains three isoforms [67, 69–71].

In pathologic states, hyperphosphorylation of tau weakens the interaction between tau and the microtubule, causing tau to dissociate from the microtubule and form into insoluble aggregates [69, 72]. The composition of these aggregates depends on the ratio of tau isoforms, the degree of hyperphosphorylation, and multiple other posttranslational modifications [73]. Morphologically, these aggregates may consist of paired helical filaments, straight filaments, or twisted filaments [74–78]. The neuropathologic changes in Alzheimer’s disease include the formation of neuronal tau aggregates consisting of a mixture of 3R and 4R tau isoforms, which form into paired helical filaments and straight filaments [79, 80]. In non-Alzheimer’s disease tauopathies, tau inclusions are associated with tau protofilaments that have unique, disease-specific atomic structures [81]. As such, the histopathological findings can vary widely between 3 and 4R tauopathies, and between sporadic and inherited tauopathies secondary to *MAPT* mutations [82, 83]. Although the neuropathology of tauopathies may vary, neuronal and glial (both astrocytic and oligodendroglial) cytoplasmic tau inclusions are the neuropathological hallmarks of disease seen in all cases of tau-related neurodegenerative disorders [68, 70].

FTLD can result from multiple neuropathologically distinct tauopathies associated with 3R, 4R, or mixed 3R/4R tau isoforms. In addition, mutated forms of tau protein aggregate in inherited tauopathies are associated with *MAPT* mutations that lead to a change in the primary structure of tau. Pick’s disease (PiD) is a sporadic tauopathy characterized pathologically by the pathognomonic rounded intracytoplasmic inclusions (Pick bodies) composed of 3R tau [84, 85]. Tau astrocytic inclusions, named ramified astrocytes, are typical of Pick’s disease. Ballooned neurons (Pick cells) and tau oligodendroglial inclusions (coiled bodies) are also found; however, these are not specific features of Pick’s disease [85]. The most common clinical syndromes associated with Pick’s disease are bvFTD [53], nfvPPA, and svPPA [86].

The 4R tauopathies are comprised of progressive supranuclear palsy (PSP), corticobasal degeneration (CBD), globular glial tauopathy (GGT), and argyrophilic grain disease (AGD). Progressive supranuclear palsy is a pathological entity primarily affecting subcortical regions such as the midbrain, subthalamic nucleus, globus pallidus, and dentate nucleus of the cerebellum, and variable disease spreading across various neocortical regions, particularly within the frontal lobe. The pathognomonic pathological hallmarks are tufted astrocytes. Neuronal cytoplasmic inclusions acquiring a globose shape (globose neurofibrillary tangles of PSP) are also seen in regions particularly vulnerable to degeneration, such as the globus pallidus and subthalamic nucleus. Oligodendroglial coiled bodies are commonly seen in the affected cortical and subcortical white matter [87]. The classic clinical presentation, Richardson syndrome (PSP-RS), manifests as atypical parkinsonism with axial rigidity and postural instability leading to falls, bradykinesia, vertical supranuclear gaze palsy, and a dysexecutive cognitive syndrome [88]. Other FTD syndromes may arise from PSP neuropathology, including bvFTD [53], nfvPPA [86], and CBS [89].

Corticobasal degeneration is a pathological entity often associated with asymmetric cortical degeneration and characterized pathologically by 4R tau-immunoreactive astrocytic plaques and thread-like neuritic pathology in gray and white matter. Neuronal cytoplasmic inclusions, ballooned neurons, and coiled bodies are typically present together with substantia nigra neuronal loss [90, 91]. The classic clinical syndrome associated with CBD, CBS, often presents with limb or axial rigidity, bradykinesia, dystonia, cognitive and behavioral impairment, limb apraxia, cortical sensory loss, and alien limb syndrome [92]. However, it is important to remember that CBS is only associated with CBD pathology in about a third of cases [92], with most cases being caused by other neuropathological entities, mainly AD and PSP neuropathologies. Additionally, CBD can result in other FTD clinical syndromes, including bvFTD [53, 93], nfvPPA [86, 93], and PSP-S [93].

Argyrophilic grain disease (AGD) is tauopathy characterized neuropathologically by 4R tau-positive spindle-shaped inclusions in neuronal dendrites and axons (argyrophilic grains), pre-neurofibrillary tangles in neurons, and coiled bodies in oligodendrocytes [94]. Ballooned neurons may also be found similar to those in Pick’s disease and CBD, however, ballooned neurons are localized predominantly to limbic regions in AGD, whereas in Pick’ disease and CBD, they are also found in the frontal and parietal cortices [95]. GGT is characterized pathologically by 4R tau-positive globular astrocytic and oligodendroglial inclusions [96]. This rare pathological entity is associated with multiple clinical syndromes, though more commonly with bvFTD and bvFTD-MND [96–98].

### aFTLD-U

Atypical frontotemporal lobar degeneration with ubiquitin-positive inclusions (aFTLD-U) is a relatively rare cause of FTD characterized by FUS-positive inclusions, and is the underlying pathology in about 5% of all bvFTD cases [99]. In cases of aFTLD-U, the age of onset is very early, often occurring before 40 years. While patients typically meet clinical criteria for bvFTD, the clinical presentation is distinct from other variants of bvFTD, and is characterized by severe progressive psychobehavioural abnormalities, often with severe disinhibition, apathy, compulsions, and aggressive behavior [53, 100]. Cognitive dysfunction, aphasia, and motor features are less common at the time of the initial presentation [100].

Cases of aFTLD-U demonstrate severe degeneration of the frontal and temporal lobes, hippocampal CA1 and subiculum, striatum, globus pallidus, and substantia nigra [99]. It is characterized histopathologically by small, round, neuronal cytoplasmic inclusions that are immunoreactive for FUS [99, 100].

### Genetics

Frontotemporal dementia is predominantly a sporadic disease; however, about 30–40% of cases have a strong familial history [101, 102], though heritability varies by the clinical syndrome [102]. About 15% of FTD cases demonstrate an autosomal dominant pattern of inheritance [102], with the majority being due to a pathogenic expansion in *chromosome 9 open reading frame 72* (*C9ORF72*), or a mutation in *microtubule-associated protein tau (MAPT*), or *progranulin* (*GRN*) genes [102, 103]. *C9ORF72* pathogenic expansion is associated with a unique form of FTLD, which, together with features of FTLD-TDP type A or B or often unclassifiable as intermediate between type A and B, present unique pathological hallmarks in forms of intranuclear RNA-foci and cytoplasmic ubiquitinated dipeptide repeat (DPR) inclusions [104]. *C9ORF72* pathogenic expansion is more commonly associated with bvFTD, MND, or bvFTD-MND. *GRN* is invariably associated with FTLD-TDP type A neuropathology and can lead to bvFTD, nfvPPA, and CBS [86, 105–107]. Finally, the various pathogenic *MAPT* mutations are associated with distinct tauopathies that may be predominately characterized by 3R, 4R, or a mixture of 3R and 4R inclusions. With the exception of the intronic mutation and silent mutations, the *MAPT* mutation that change the primary structure of tau leads to the deposition of mutant tau species. The neuropathology associated with these cases, while often resembling prototypical CBD, PSP, or Pick’s disease, should be regarded as fundamentally different from the one observed in sporadic tauopathies.

## Neuroimaging of Frontotemporal Dementia Syndromes

### Neuroimaging Modalities

Neuroimaging modalities used in assessing neurodegenerative disorders fall into broad categories, structural, functional, and molecular imaging. Structural imaging modalities commonly used include computed tomography (CT) and structural magnetic resonance imaging (MRI), which allow for the visualization of neuroanatomy. Functional imaging encompasses a broad variety of modalities such as positron emission tomography (PET), single-photon emission computed tomography (SPECT), and functional MRI (fMRI), which measure various parameters such as metabolic activity, regional blood flow, or hemodynamic changes while the patient is either at rest or performing a specific task. In addition, molecular imaging refers to techniques that measure molecular and cellular events in living organisms (e.g., specific receptors or protein aggregates). Of all neuroimaging tools, structural MRI and PET are two of the most commonly used in the assessment of FTD, both in the clinical and research setting. As such, this review will focus primarily on these two techniques.

#### Magnetic Resonance Imaging

MRI works by utilizing a strong magnet that forces protons in the body to align parallel or antiparallel to the magnetic field vector. A radiofrequency pulse is applied, which disrupts the alignment of protons along the magnetic field vector, causing them to spin out of equilibrium. The radiofrequency pulse is then stopped, and the protons realign with the magnetic field, releasing energy in the process. The length of time that it takes for protons to realign with the magnetic field, and the amount of energy released during the process, can be measured and varies between tissue types. These variations can then be displayed and differentiated based on signal intensity [108].

MRI is a widely used imaging modality in both the clinical and research setting. In the clinical setting, MRIs are typically analyzed visually by a trained radiologist. However, in the research setting, automated computational techniques are commonly employed to assess brain structure. One of the most common approaches to assessing the volume of specific brain regions (“regions of interest”), determined a priori. Other approaches, such as voxel-based morphometry [109] or cortical thickness analyses [110], allow running exploratory analyses of the entire brain structure.

#### Positron Emission Tomography

Positron emission tomography (PET) is a nuclear imaging technique that allows for three-dimensional mapping of physiologic processes in vivo. PET imaging works on the principle of beta plus (β^+^) decay, a type of radioactive decay in which a proton in an unstable radioisotope undergoes conversion to a neutron, resulting in the emission of a positron and an electron neutrino. The emitted positron collides with an electron within the tissue, resulting in the annihilation of the particles and emission of two photons, which a PET scanner can detect. The PET image is then displayed as a three-dimensional image with different intensity levels corresponding to the radiotracer concentration [111, 112]. Many radiotracers have been developed to target a number of proteins, including neuroreceptors, reuptake transporters, synaptic proteins, enzymes, and aggregated proteins such as amyloid-beta and tau [113].

PET is often utilized to measure the degree of neuronal metabolic activity in vivo through the use of the radiotracer ^18^F-fluoro-2-deoxyglucose (FDG), a glucose analog labeled with an ^18^F radioisotope. FDG is readily taken up by metabolically active cells, and the degree of FDG uptake can be assessed by PET to determine the extent of cellular metabolic activity. Decreased levels of FDG uptake suggest hypometabolism of the brain region being assessed, indicating potential dysfunction. Although FDG-PET only characterizes the relative metabolism of brain regions and does not provide direct information regarding the etiology of disease, regional patterns of hypometabolism can help identify specific neurodegenerative diseases and underlying pathology [111, 112].

PET radiotracers have been developed to visualize pathological proteins in vivo. Much of the research regarding neurodegenerative diseases has focused on small-molecule PET radiotracers that bind to amyloid-beta or tau. The first radiotracer selective for Aβ, [^11^C]Pittsburgh Compound-B (PiB), was developed in the early 2000s at the University of Pittsburgh [114, 115]. The Pittsburgh group demonstrated that small-molecule PET radiotracers were effective in characterizing the burden of amyloid beta in vivo [116], paving the way for the development of additional small-molecule PET radiotracers, including [^18^F]Florbetapir and [^18^F]Florbetaben, which have been used extensively in the research setting to characterize Alzheimer’s disease [117].

About a decade after PiB was developed, PET radiotracers targeting tau with a high degree of specificity were demonstrated. To date, most studies have utilized first-generation tau radiotracers, namely [^18^F]Flortaucipir (formerly known as AV1451 or T807), [^18^F]THK5117, [^18^F]THK5351, and [^11^C]PBB3. These radiotracers readily bind to intracellular and extracellular tau, neuritic plaques, and ghost tangles [118, 119]; however, off-target binding to molecules other than tau is a major issue with first-generation radiotracers [118, 119]. Numerous second-generation tau radiotracers have been developed to improve the binding specificity to tau and have reduced the degree of off-target binding [119].

### Typical Neuroimaging Features of the Core FTD Phenotypes

**Fig. 1:**
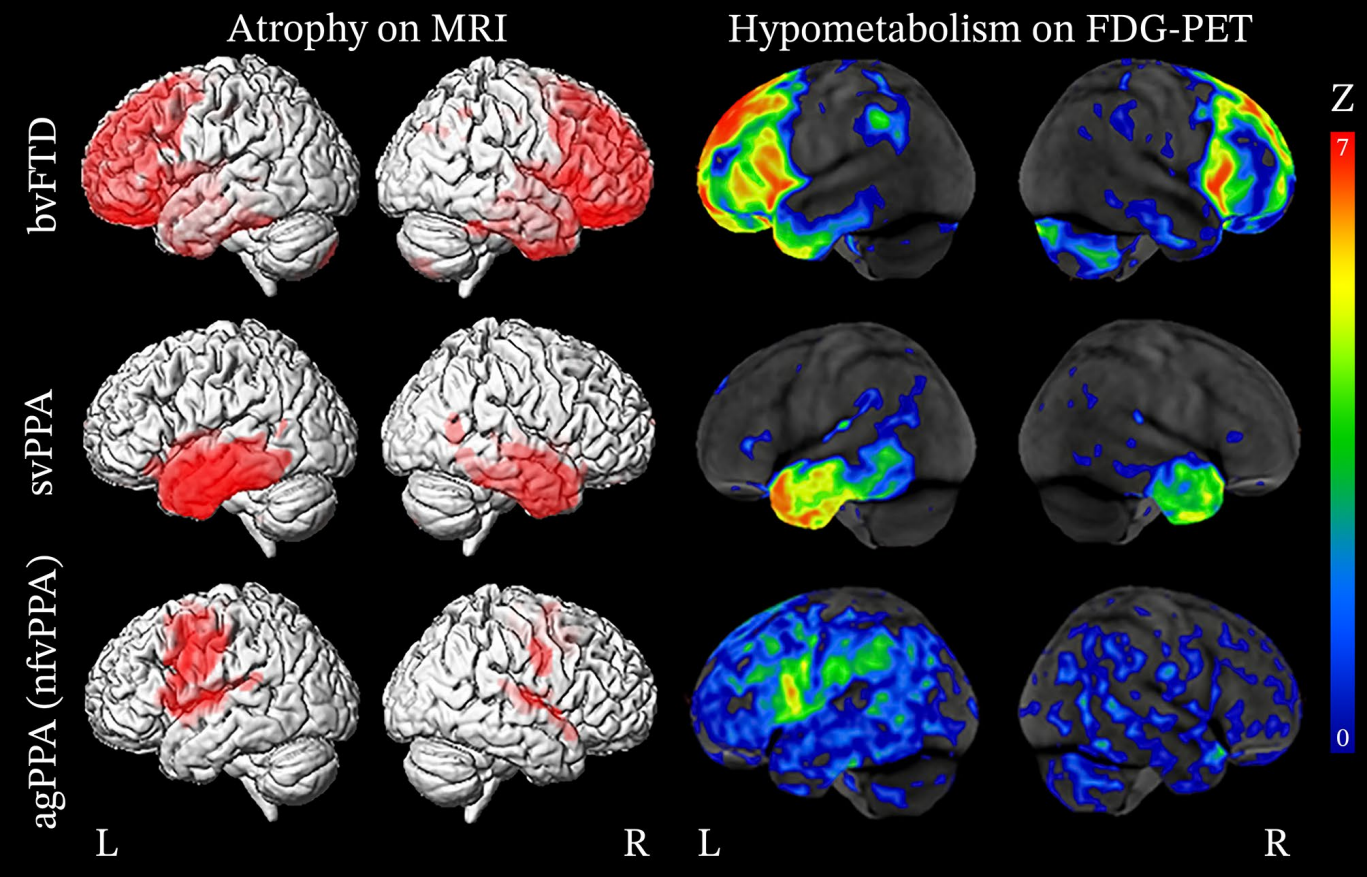
Patterns of atrophy and hypometabolism associated with FTD clinical syndromes. Group-level maps depicting gray matter atrophy compared to controls are shown on three-dimensional renders of the brain. Hypometabolism on FDG-PET is shown for individual patients, represented as Z score maps depicting abnormalities compared to controls. Adapted with permission from Whitwell 2019 [258]

This section describes the general patterns of atrophy and hypometabolism described in patients grouped based on their clinical diagnoses (Fig. 1).

#### Behavioral Variant Frontotemporal Dementia

Behavioral variant FTD is associated with degeneration of the prefrontal cortex (PFC), anterior temporal lobes, limbic, and subcortical regions [120, 121]. Early in the disease course, von Economo neurons in the anterior insula and anterior cingulate cortex are selectively vulnerable [122]. These regions represent the epicenter of pathology in bvFTD and are often the first to demonstrate structural and metabolic neuroimaging abnormalities [123–125]. Pathological protein aggregates are thought to spread to other regions by propagating along brain networks in a prion-like manner, leading to disease progression [123–129]. In general, atrophy is asymmetric and typically involves the right hemisphere more than the left as demonstrated on structural neuroimaging [53, 130]. However, a recent study by Irwin et al. [131] examining the asymmetry of post-mortem neuropathology in bvFTD showed that the while the right orbitofrontal cortex was generally more atrophic than the left, the left ventrolateral temporal lobe was generally more atrophic than the right, particularly in cases of FTLD-tau [131].

Structural MRI may reveal atrophy of the anterior insula and anterior cingulate cortex in the earliest stages of the disease before patients fulfill the criteria for a diagnosis of bvFTD [121, 124, 132–139]. As the disease progresses into the mild stages, additional areas become increasingly involved. The frontal lobes are widely impacted and typically exhibit degeneration of the frontal poles, dorsolateral PFC, medial PFC, orbitofrontal cortex, and premotor cortex [134–137, 140]. The anterior temporal lobes, limbic structures, including the hippocampus and amygdala, as well as the thalamus and striatum, are also commonly impacted [135–138, 140]. In the later stages of the disease, atrophy becomes more pronounced and involves the contralateral hemisphere to a greater degree, widely affecting the frontal and temporal lobes [133, 138, 140–142]. Atrophy may also extend posteriorly to involve the parietal lobes and cerebellum [140, 141].

In cases where structural imaging is equivocal, FDG-PET can potentially help determine if there is underlying neuronal dysfunction. FDG-PET typically demonstrates hypometabolism of the frontal and temporal lobes and is generally spatially consistent with atrophy patterns, with larger effect sizes [143]. Hypometabolism can often be found impacting the frontal lobes, particularly in the medial PFC, dorsolateral PFC, ventrolateral PFC, orbitofrontal cortex, and anterior cingulate cortex [138, 144, 145]. The anterior and inferior aspect of the temporal lobes are commonly impacted and extends to the posterior fusiform gyrus [138, 145]. The medial temporal lobe is also commonly involved and hypometabolism of associated limbic structures, including the hippocampus and amygdala [138, 145], as well as the caudate and thalamus [144, 145]. In the later stages of the disease, further hypometabolism is demonstrated in the regions initially impacted [138, 142, 146], as well as the frontal poles, supplementary motor area, middle temporal gyri, posterior cingulate cortex, precunei, inferior parietal lobes, lateral superior occipital cortex, and cerebellum [138, 146].

#### Semantic Variant Primary Progressive Aphasia

The hallmark of svPPA on structural MRI is asymmetric degeneration of the anterior temporal lobes, impacting the language-dominant cerebral hemisphere (typically the left) more than the non-language dominant hemisphere. In the early stages of disease, atrophy typically involves the medial temporal lobe, specifically the hippocampus, amygdala, fusiform gyrus, and the inferior and middle temporal gyri, which exhibit a strong anterior–posterior gradient [147]. Atrophy can also be observed in the medial PFC, orbitofrontal cortex, pars opercularis, insula, and striatum [138, 148]. As the disease progresses, brain involvement becomes increasingly diffuse with more widespread atrophy of the frontal lobes, parietal lobes, and to a lesser degree, the occipital lobe. Specific areas of involvement include the superior and middle frontal gyrus, anterior cingulate cortex, posterior cingulate cortex, parietal operculum, precuneus, angular gyrus, supramarginal gyrus, superior parietal lobule, occipital fusiform gyrus, lingual gyrus, and the superior and inferior lateral occipital cortex [138].

Metabolic imaging with FDG-PET reveals hypometabolism in regions where atrophy is typically demonstrated on structural imaging [149, 150]. The most significant degree of hypometabolism is seen in the inferior, middle, and superior regions of the anterior temporal lobes, extending posteriorly to the temporoparietal junction, more so on the left than the right [151–154]. Other affected regions include the orbitofrontal cortex, anterior cingulate cortex, insula, amygdala, subiculum, entorhinal cortex, and hippocampus [154, 155].

In a study by Bejanin et al. [138], FDG-PET revealed diffuse hypometabolism throughout the brain in the mild stages of disease (Clinical Dementia Rating (CDR) 0.5), sparing only the occipital lobes. Specifically, hypometabolism was noted in the frontal lobes (frontal pole, orbitofrontal cortex, medial PFC ventrolateral PFC, dorsolateral PFC, and frontal operculum), temporal lobes (inferior temporal gyrus, middle temporal gyrus, superior temporal gyrus, planum polare and temporale, transverse temporal gyrus, and fusiform gyrus, extending posteriorly to the temporoparietal junction), parietal lobes (angular gyrus, supramarginal gyrus, and parietal operculum), and subcortical structures (anterior cingulate cortex, insula, hippocampal complex, striatum, and thalamus). Interestingly, hypometabolism was present in regions without atrophy, including the frontal poles, dorsolateral PFC, pars triangularis, angular gyrus, supramarginal gyrus, parietal operculum, and the thalamus. In later stages of disease (CDR 1–2), additional areas were increasingly involved, including the precentral and postcentral gyri, supplementary motor area, superior frontal gyrus, posterior cingulate cortex, and superior parietal lobule, all of which demonstrated a significantly greater degree of hypometabolism than would be expected given the degree of atrophy.

#### Nonfluent/Agrammatic Variant Primary Progressive Aphasia

The region of the earliest involvement in nfvPPA is found in the left inferior frontal gyrus (pars triangularis and pars opercularis) [124, 148, 156]. Additional areas of atrophy observed early in the disease course include the insula, supplementary motor area, premotor cortex, and motor cortex [152, 157]. Other areas of involvement during the disease course include the medial PFC, dorsolateral PFC, orbitofrontal cortex, medial and lateral temporal lobe, striatum, and thalamus [138, 157–159]. Additionally, some studies have demonstrated that compared to individuals with mixed agrammatism and apraxia of speech, those who exhibit predominantly agrammatism have more pronounced atrophy and hypometabolism in the prefrontal and anterior temporal lobes [46–49, 160].

Hypometabolic regions seen with FDG-PET are most significant in the left ventrolateral PFC, including the pars opercularis and pars triangularis, dorsolateral PFC, medial PFC, and supplementary motor area [138, 152]. Other regions where hypometabolism is observed include the orbitofrontal cortex, anterior cingulate cortex, insula, precentral and postcentral gyrus, parietal operculum, striatum, and thalamus [152].

## Neuroimaging of FTLD Neuropathologic Subtypes

Numerous studies have demonstrated specific patterns of atrophy correlating with the various neuropathologic subtypes of FTLD-TDP and FTLD-tau. In clinical syndromes associated with multiple neuropathologic subtypes, such as bvFTD, the different atrophy and metabolic patterns of each subtype can help narrow the differential diagnosis and predict the underlying neuropathology. Importantly, genetic mutations can substantially impact the pattern of atrophy across the FTLD subtypes. Genetics will be discussed briefly, however, for a more in-depth discussion regarding the role of genetic mutations in FTLD, we recommend the recent review by Häkkinen et al. [161].

### FTLD-TDP

#### FTLD-TDP Type A

**Fig. 2:**
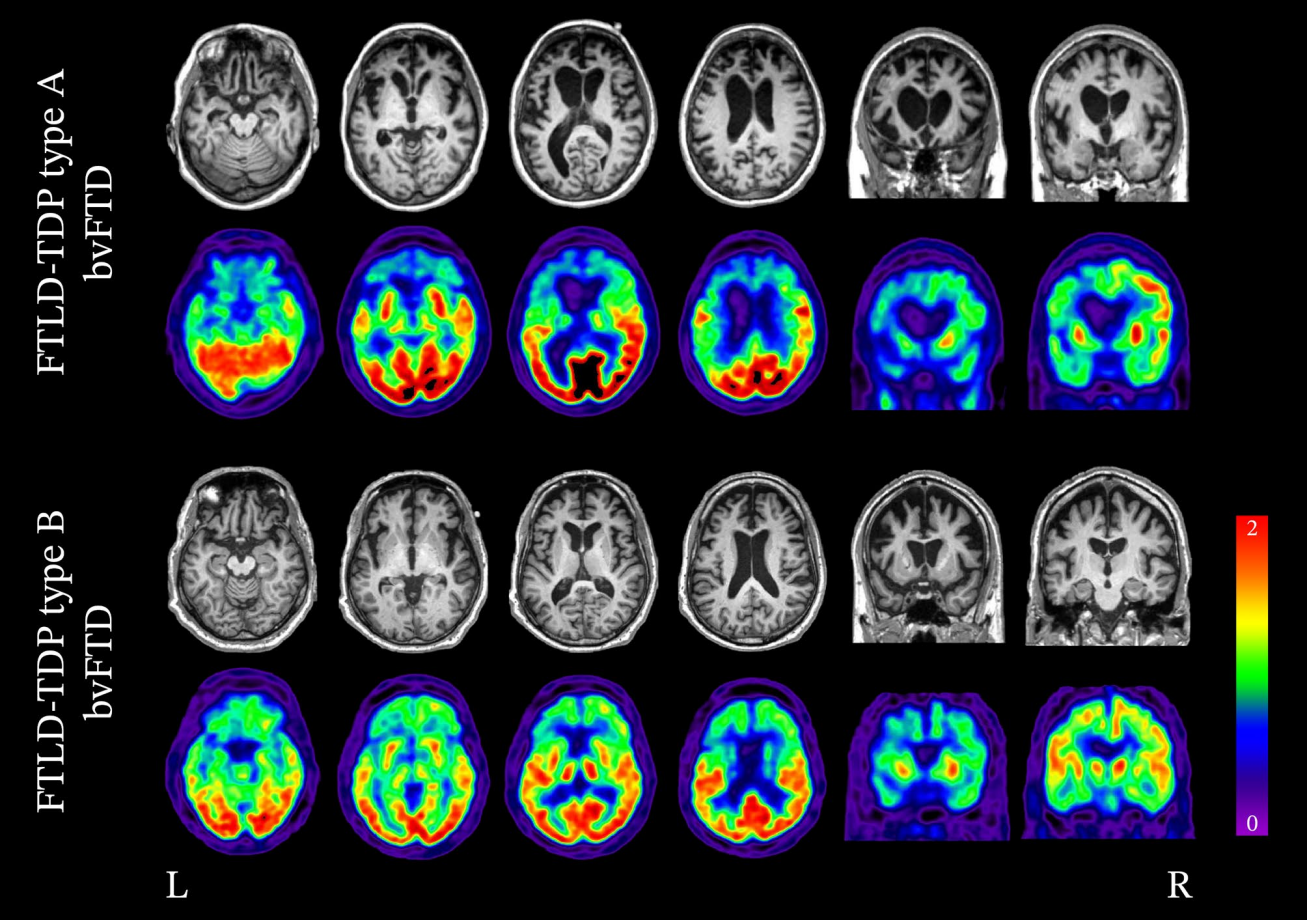
Neuroimaging of bvFTD associated with FTLD-TDP type A and B (top row: structural MRI; bottom row: FDG-PET). The variability in patterns of degeneration attributed to FTLD-TDP neuropathology is readily seen in these two cases of bvFTD. In the case of bvFTD associated with FTLD-TDP type A, degeneration and hypometabolism of the bilateral frontal lobes are present; however, the left is significantly more impacted than the right and extends to the left parietal lobe. In bvFTD associated with FTLD-TDP type B, significant degeneration of the bilateral frontal lobes is seen; however, in contrast to type A, the parietal lobes are less affected and atrophy is relatively symmetrical

FTLD-TDP type A is associated with a variety of clinical presentations, namely bvFTD, nfvPPA, and to a lesser extent, CBS. In imaging studies of patients with FTLD-TDP type A, atrophy and hypometabolism has been found to be widespread and asymmetric (either left- or right-predominant), involving the frontal, temporal, and parietal lobes [162, 163]. A number of retrospective analyses exploring clinicopathologic and neuroimaging features in patients with FTLD-TDP type A have found similar patterns of atrophy. The most significantly impacted regions include the dorsal frontal lobes, anterior, medial, and posterior temporal lobes, inferior parietal lobes, orbitofrontal cortex, insula, frontal operculum, caudate, and thalamus (Fig. 2) [53, 89, 162, 163]. There is particular involvement of the anterior insula, frontal operculum, and parietal lobes, which can help differentiate FTLD-TDP type A from other FTLD-TDP subtypes [53, 89, 163]. Among the various FTLD pathological entities, FTLD-TDP type A is the strongest association with hippocampal sclerosis.

Although FTLD-TDP type A often occurs sporadically, it is also the prototypical neuropathology of inherited FTLD associated with *GRN* mutations. Mutations in *GRN* often result in a haploinsufficiency of the progranulin protein and progressive neurodegeneration [60, 164–167].

Atrophy is typically widespread and asymmetric. A 2015 study by Rohrer et al. [168] involving 45 asymptomatic *GRN* carriers from the Genetic Frontotemporal Dementia Initiative (GENFI) cohort observed atrophy of the insula 15 years before expected symptom onset, in the temporal and parietal lobes 10 years before expected onset, and the striatum 5 years before expected onset. With progression of disease, atrophy increasingly impacts the dorsolateral PFC, ventromedial PFC, insula, anterior cingulate cortex, superior and lateral temporal lobes, striatum, and lateral and medial parietal lobes [164, 169, 170]. Atrophy of the medial parietal lobes is particularly characteristic of GRN mutations and may help differentiate *GRN* from other genetic subtypes [107, 164, 169, 170].

#### FTLD-TDP Type B

FTLD-TDP type B is typically associated with bvFTD and FTD-MND [51, 60, 171–173]. Atrophy and hypometabolism are relatively symmetric and involve the medial and lateral frontal lobes (Fig. 2) [53, 162, 163]. Limbic and subcortical structures are also significantly involved, namely the anterior cingulate, anterior insula, hippocampi, striatum, and thalamus [53, 162, 163]. At a group level, atrophy of the parietal lobes is more pronounced in FTLD-TDP type B when compared to type C. Additionally, type B has the least amount of temporal lobe atrophy when compared to the other FTLD-TDP subtypes [163].

The most common genetic cause of FTD-MND is a hexanucleotide repeat expansion within the *C9ORF72* gene, which is also the most common genetic cause of FTD, and of amyotrophic lateral sclerosis (ALS) [174, 175]. *C9ORF72* most commonly results in FTLD-TDP type B pathology, though type A pathology also occurs to a lesser degree [176]. The presence of a neuropathology with mixed type A and type B features, or unclassifiable features is also common. The most distinctive feature of FTLD-TDP linked to *C9ORF72* remains the presence of DPR cytoplasmic inclusions and intranuclear RNA foci inclusions. These inclusions are thought to precede TDP-43 deposition and are the only pathological hallmarks found in some cases [177]. Among *C9ORF72* carriers who develop a neurodegenerative disease, bvFTD occurs in about 48%, FTD-MND in about 36%, and ALS in about 16% [176]. Neuroimaging often reveals early atrophy and hypometabolism of subcortical structures, including the thalamus, hippocampus, amygdala, and striatum. Cortical atrophy and hypometabolism are often mild, but widespread and symmetric. Regions commonly found to be involved include the orbitofrontal cortex, dorsolateral PFC, ventromedial PFC, anterior temporal lobes, anterior and posterior cingulate cortex, superior and inferior parietal lobes, and cerebellum. Impacted regions that help differentiate cases of *C9ORF72* from other genetic subtypes include the thalamus, inferior parietal lobe, and superior cerebellum [161, 170, 176]. In a group of 16 patients, including a majority of patients with FTLD-TDP type B pathology and 7 cases with a *C9ORF72* mutation, Pasquini et al. showed that the severity of TDP pathology measured in von Economo neurons and fork cells in the right frontal insula was correlated with the severity of antemortem atrophy in the salience network [178].

#### FTLD-TDP Type C

**Fig. 3:**
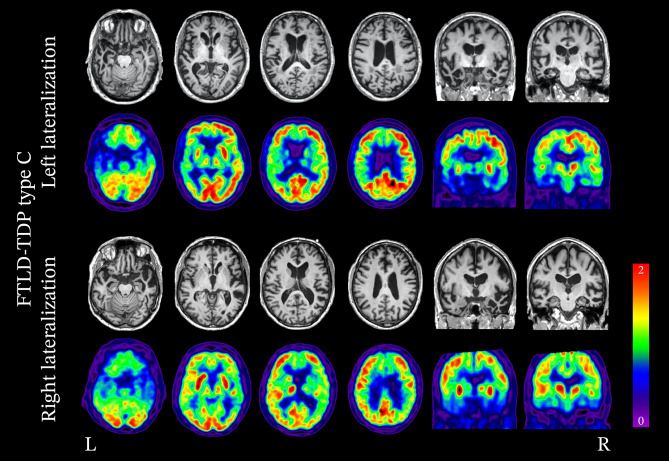
Neuroimaging patterns associated with FTLD-TDP type C. FTLD-TDP type C typically exhibits significant anterior temporal lobe degeneration, which strongly lateralizes to either the left or right hemisphere. In cases of left lateralization, the clinical syndrome is typically consistent with svPPA, whereas in cases of right lateralization, there is a greater degree of behavioral symptoms, and language deficits are not typically the initial symptoms

**Fig. 4:**
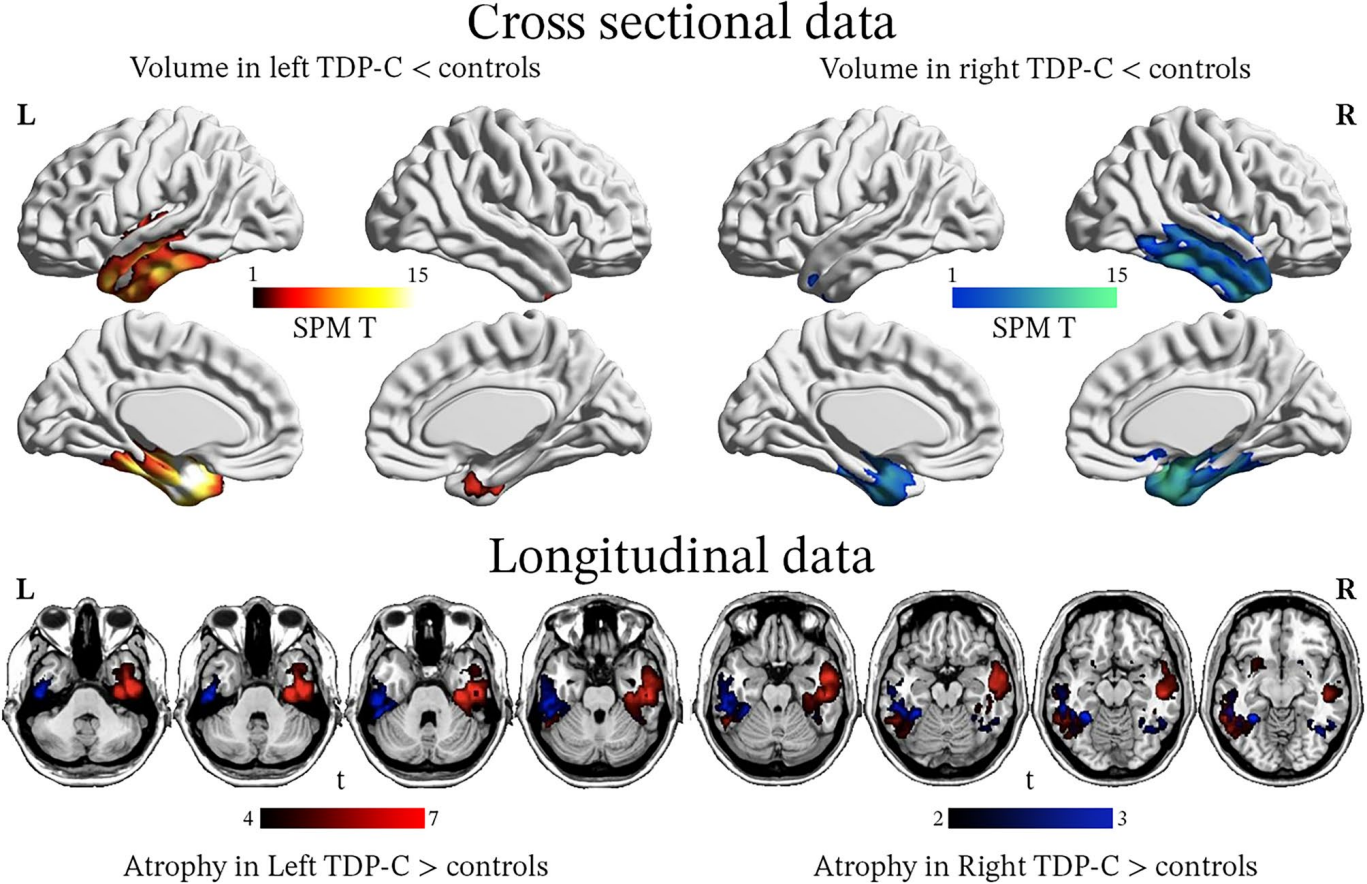
Gray matter atrophy associated with FTLD-TDP type C: left and right variants. All patients had pathology-proven TDP-C but were classified as having either a left- (*n* = 18) or right- (*n* = 12) predominant pattern of atrophy on ante mortem MRIs. At baseline, both groups show asymmetric but bilateral volume reduction in the temporal lobes, with a strong predominance in anterior and medial areas. In patients with multiple MRIs (*n* = 13 left- and 4 right-predominant cases), longitudinal analyses revealed that in both subgroups, atrophy progressed to the contralateral hemisphere and to more posterior temporal areas. Adapted with permission from Borghesani 2020 [179]

FTLD-TDP type C is strongly associated with asymmetric, degeneration of the medial aspect of the anterior temporal lobes, specifically the temporal pole, fusiform gyrus, lingual gyrus, and amygdalo-hippocampal area (Fig. 3) [179, 180]. In the majority of cases where imaging abnormalities are predominant in the language-dominant (most commonly the left hemisphere) temporal lobe, the clinical diagnosis is svPPA [53, 163]. The retrospective study of 30 patients with FTLD-TDP type C pathology and available ante mortem MRI from Borghesani et al. showed that 12 cases (40%) demonstrated lateralization of atrophy to the non-language dominant hemisphere [179]. This pattern was associated with the onset of behavioral symptoms preceding language impairment [179, 181, 182]. Regardless of whether the disease is initially lateralized to the right or left hemisphere, atrophy progresses in a stereotypical pattern [179]. Initially, it can be detected in one of the anterior temporal lobes, then progresses to both the ipsilateral posterior temporal areas and the contralateral temporal pole (Fig. 4), and later involves the orbitofrontal cortex. However, the greatest degree of atrophy is found in the anterior temporal lobes throughout the disease course [179].

### FTLD-Tau

#### The Three-Repeat Tauopathy Pick’s Disease

**Fig. 5:**
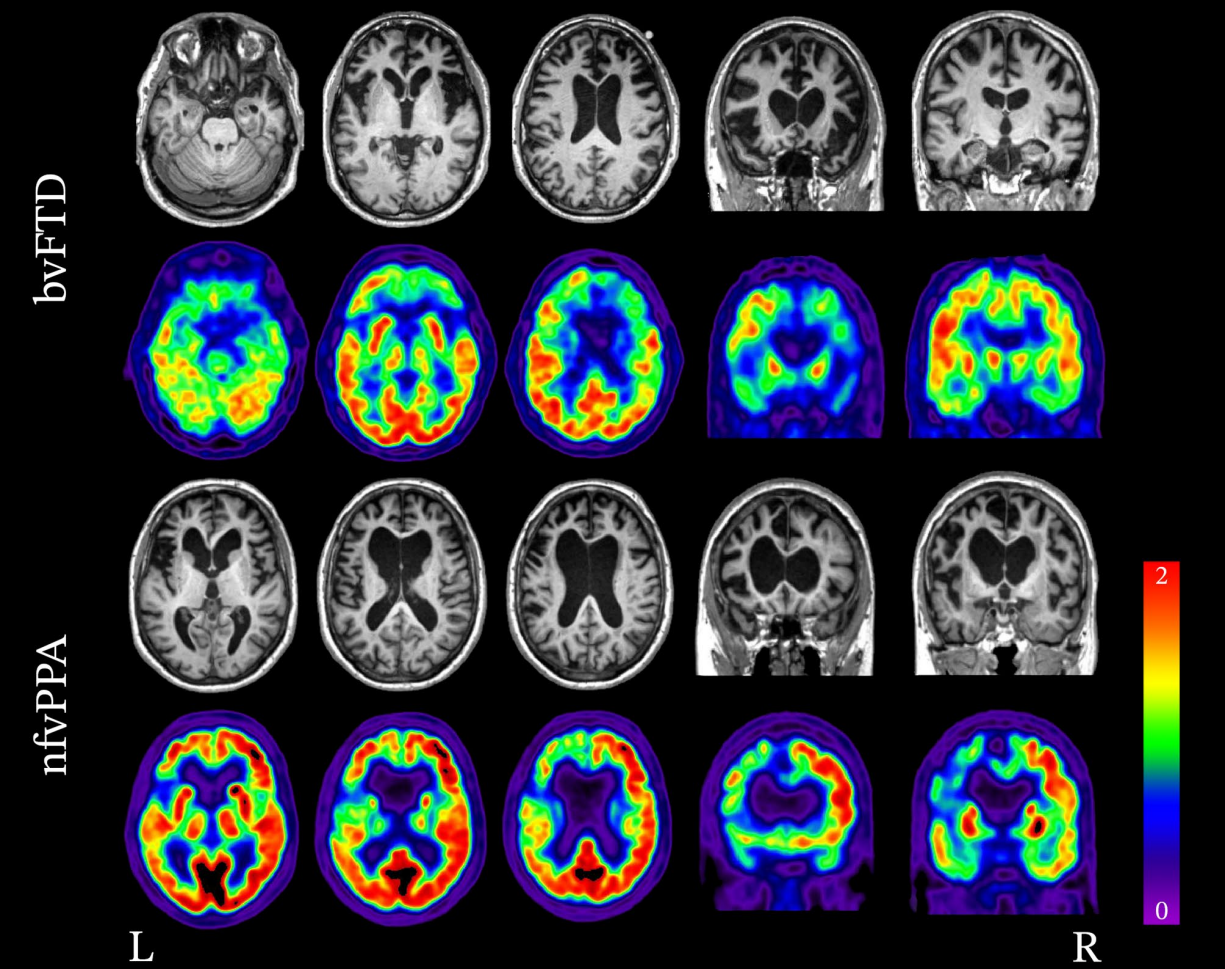
Neuroimaging patterns associated with Pick’s disease (FTLD-tau, 3R). Structural MRI and FDG-PET demonstrating the variability in patterns of atrophy and hypometabolism attributed to Pick’s disease (3R tau) neuropathology. In the case of bvFTD, significant bilateral frontal lobe atrophy and hypometabolism is seen. In the case of nfvPPA, atrophy and hypometabolism is lateralized and is greatly impacting the left frontal lobe more so than the right.

Pick’s disease (PiD) is the only sporadic 3R tauopathy FTLD. It is primarily associated with bvFTD, though nfvPPA, svPPA, and CBS can also occur [183–185]. In general, neuroimaging in PiD demonstrates striking atrophy, often described as “knife-edge” due to its severity (Fig. 5), affecting the frontal and temporal lobes, specifically the dorsolateral PFC, ventromedial PFC, temporal poles, and the anteromedial and anterolateral regions of the temporal lobes, with often remarkable sparing of the peri-Rolandic gyri. Subcortical structures, including the anterior cingulate cortex, anterior insula, posterior insula, and caudate, are also impacted, while the substantia nigra is fairly spared from neurodegeneration compared to the degree observed in 4R tauopathies such as CBD and PSP [186–188].

In cases of bvFTD, circumscribed frontotemporal atrophy is present throughout the frontal and anterior temporal lobes [187]. In a recent study by Whitwell et al. [189] of 17 individuals with PiD (8 with bvFTD, 6 with nfvPPA, 1 with svPPA, 1 with unclassified PPA, and 1 with CBS), those with bvFTD showed a striking degree of predominantly right-sided atrophy involving the insula, anterior cingulate cortex, and orbitofrontal cortex. Over time, atrophy increasingly involved the frontal poles, anterior and middle cingulate gyri, medial frontal lobe, gyrus rectus, orbitofrontal cortex, inferior and middle frontal gyri, temporal poles, inferior and middle temporal lobes, fusiform gyrus, parahippocampal gyrus, right anterior hippocampus, precuneus, right angular and supramarginal gyri, and bilateral basal ganglia. Similar findings have been described in other studies [53, 89, 187, 188].

In cases of nfvPPA, the left inferior frontal gyrus, middle frontal gyrus, insula, and supplemental motor area are significantly atrophic. Other regions of atrophy include the precentral gyrus and orbitofrontal gyrus bilaterally [86, 189].

Semantic variant PPA can also be associated with PiD, although very rarely. A study by Spinelli et al. [86] of 29 individuals with svPPA, of which only two had PiD, found that the anterior cingulate cortex and striatum were affected to a greater degree in PiD compared to that seen in FTLD-TDP type C. Additionally, frontal lobe and subcortical atrophy was more substantial when compared to other pathologies associated with svPPA [86]. Specific regions most impacted in those with svPPA due to PiD include the bilateral anterior temporal lobes, orbitofrontal cortex, inferior frontal gyrus, middle frontal gyrus, superior frontal gyrus, anterior cingulate cortex, insula, striatum, left middle temporal gyrus, and fusiform gyrus [86].

#### Four-Repeat Tauopathies

Multiple 4R tauopathies may lead to FTD, including PSP, CBD, GGT, and AGD. PSP and CBD are, by far, the most common 4R tauopathies resulting in FTD syndromes. The epicenters of PSP neuropathology are heterogeneous, leading to a variety of clinical syndromes associated with PSP neuropathology. PSP most commonly presents as Richardson syndrome (PSP-RS), although it may manifest as parkinsonism (PSP-P), pure akinesia with gait freezing (PSP-PAGF), CBS (PSP-CBS), bvFTD (PSP-bvFTD), or nfvPPA (PSP-nfvPPA). The degree of brainstem degeneration is greater in PSP-RS, PSP-P, and PSP-PAGF and is associated with pronounced atrophy of the midbrain and superior cerebellar peduncles, which are characteristic findings on structural MRI [190, 191]. Additionally, several neuroimaging findings have been proposed as suggestive of PSP pathology, including the “hummingbird sign,” “Mickey Mouse sign,” and “morning glory sign.” However, their sensitivity in clinical practice is limited [192, 193]. The hummingbird sign is seen on midline sagittal T1 sequences as atrophy of the midbrain, resulting in a flattening or concavity of the superior aspect of the midbrain, which is normally convex [194, 195]. The Micky Mouse sign refers to a reduction in the anterior–posterior diameter of the midbrain at midline when viewed in the axial plane [195, 196]. The morning glory sign refers to a concavity of the lateral margin of the tegmentum of the midbrain when viewed in the axial plane [197].

**Fig. 6:**
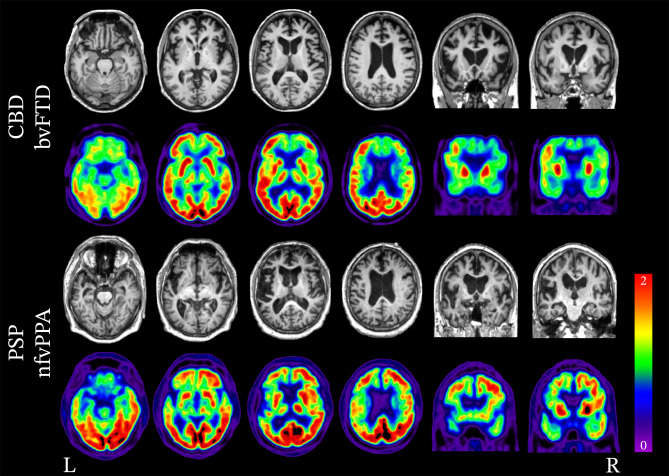
Neuroimaging patterns associated with 4R tauopathies (FTLD-tau). Structural MRI and FDG-PET demonstrating the variability in patterns of atrophy and hypometabolism attributed to 4R tauopathies, CBD and PSP

In cases of PSP-bvFTD, frontal atrophy is often relatively mild, and the temporal lobes are almost always spared, whereas posterior cerebellar atrophy is prominent and might help distinguish PSP-bvFTD from other subtypes [53]. Atrophy in CBD-bvFTD (Fig. 6) tends to be more frontal than that seen in PSP. It involves the dorsolateral and medial frontal regions, with involvement of both the precentral and postcentral gyri, distinguishing CBD-bvFTD from other subtypes [53]. In cases of nfvPPA, the left inferior frontal gyrus is the epicenter of disease and is atrophic in both PSP and CBD. PSP-nfvPPA exhibits a greater degree of atrophy in the bilateral precentral and middle frontal gyri, supplemental motor area, dorsal midbrain, and right cerebellar regions compared to CBD-nfvPPA. In CBD-nfvPPA, atrophy is distributed more widely and found in the left insula, putamen, supplemental motor area, and the left precentral, middle, and inferior frontal gyri [198]. In a group of 26 patients with underlying 4R tau pathology (PSP or CBD) and various clinical diagnoses (including bvFTD), Spina et al. [199] showed strong associations between grey matter volumes measured on antemortem MRI and neuropathological measures at autopsy. More specifically, they showed that higher scores of tau inclusion burden and neurodegeneration (a composite measure of neuronal loss, astrogliosis, and microvacuolations) were partly independently predictive of lower grey matter volumes.

FTLD-tau caused by *MAPT* mutation is overall characterized by early and severe symmetric bilateral temporal lobe degeneration, with early involvement of the anterior cingulate gyrus, orbitofrontal, and fronto-insular regions. Early degeneration of the hippocampal formation is common [83, 200].

### Atypical Frontotemporal Lobar Degeneration with Ubiquitin-Positive Inclusions (aFTLD-U)

In cases of aFTLD-U, neuroimaging shows extensive atrophy of the bilateral frontal and temporal lobes [53, 201–203]. A small case series of 2 patients by Rohrer et al. [202] showed atrophy predominantly affecting the orbitofrontal cortex, insula, anteromedial temporal lobe, anterior cingulate, and caudate. An independent analysis of 3 cases with aFTLD-U showed that caudate atrophy was major and exceed the degree of atrophy seen in FTLD-TDP and FTLD-tau subtypes (*n* = 10 in each group) [203].

## Amyloid- and Tau-PET in FTD and FTLD

### Amyloid-PET to Detect AD Neuropathology

The emergence of amyloid plaque-binding radiotracers in the mid-2000s has made it possible to detect the defining features of AD neuropathology in living patients. PET-to-autopsy studies have shown that elevated amyloid-PET signal is highly specific to amyloid neuropathology: false-positive scans (i.e., high cortical PET signal in the absence of amyloid deposits) are rare [204–206]. Yet, the presence of amyloidosis is not always equivalent to clinically relevant AD neuropathology, which also requires AD tau tangles [207]: Braak III or above for intermediate level of AD neuropathology changes, Braak V or VI for high level. Moreover, amyloid-PET positivity is not always associated with clinical deficits as it is frequently found in clinically normal individuals, especially at an older age [208].

Amyloid-PET positivity is relatively rare (< 15%) in patients with a clinical diagnosis of bvFTD, svPPA, or nvfPPA [209–212], in line with autopsy studies of FTD showing a low frequency of underlying AD neuropathology (see earlier section). However, as previously mentioned, amyloid-PET positivity should be interpreted with caution, as it does not necessarily indicate that AD neuropathology is the main or sole etiology of a clinical syndrome. In cases with FTD, additional imaging information might help estimate the likelihood of underlying AD neuropathology versus “incidental” amyloidosis. Indeed, multiple groups have reported cases with amyloid-PET positivity in patients with an FTD syndrome who also showed typical FTD-like patterns of atrophy or glucose metabolism (not the posterior, temporo-parietal pattern of degeneration seen in AD). In these cases, autopsy typically showed mixed neuropathology, with the co-occurrence of FTLD and AD [213–215].

### Tau-PET in AD, FTLD-Tau, and FTLD-TDP

Radiotracers developed for tau-PET have originally been developed to detect AD-type tau pathology (i.e., paired helical filaments composed of 3R/4R tau). In vivo studies have shown that tau-PET distinguishes amyloid-positive patients with a clinical diagnosis of AD dementia from patients with FTD syndromes with high accuracy [216]. Recent PET-to-autopsy studies [217–219] have consistently reported elevated Flortaucipir-PET signal in patients with advanced AD tau pathology (usually Braak stages IV or V), and the FDA recently approved Flortaucipir for the estimation of aggregated tau neurofibrillary tangles in adult patients with cognitive impairment who are being evaluated for AD [220].

In addition to the ability to distinguish AD from other processes, several groups have used tau-PET in patients with FTD syndromes to assess its usefulness in distinguishing FTLD-tau pathology from other types of neuropathology. Early studies validating Flortaucipir on brain tissues have found little or no binding to straight filaments in non-AD tauopathies, and there is no binding to other abnormal protein deposits such as alpha-synuclein or TDP-43 [221–225]. In vivo studies have reported conflicting findings. On the one hand, Flortaucipir binding has not been observed in patients with pathology-proven FTLD-tau (e.g., Pick’s disease and CBD) [189, 217], or in groups of patients with suspected FTLD-tau (e.g., patients with nfvPPA or some patients with bvFTD) [226–229]. In these patients, Flortaucipir signal is mild (lower than what can be observed in AD) and often found in the subcortical white matter underlying frontal, cingulate, and insula cortices, and in the putamen and globus pallidus. On the other hand, similar mild Flortaucipir PET signal has been extensively reported in most patients with svPPA [227, 228, 230, 231], while the vast majority of patients are expected to harbor FTLD-TDP type C pathology, as described in previous sections. In these patients, Flortaucipir PET signal is seen in the anterior temporal lobes, in areas showing atrophy/hypometabolism. To date, the biological underpinnings of the tracer accumulation in this degenerative tissue is not understood [232, 233], but it should be noted that Flortaucipir is known to have multiple sources of “off-target” binding [119, 223, 228, 234–237].

Recently, Soleimani-Meigooni et al. published a case series of 20 patients who underwent in vivo Flortaucipir PET and autopsy, including 12 with a neuropathological diagnosis of FTLD [217]. The authors showed that a mild increase in Flortaucipir-PET binding was not a reliable indicator of FTLD-tau pathology: such signal was observed in patients with underlying 4R tau pathology (PSP, CBD) but also in a patient with bvFTD and underlying TDP-A pathology due to a GRN mutation. In this patient, tau immunohistochemistry was negative in the areas showing high Flortaucipir signal in vivo.

Finally, a few studies have used tau-PET in patients with MAPT mutations [228, 238–240]. Overall, elevated Flortaucipir-PET signal was observed in a subset of patients with specific mutations that result in a mix of 3R/4R tau pathology, namely V337M and R406W. This observation is not tracer-specific as tau-PET binding has also been observed in R406W mutation carriers with [18F]RO848, a “second generation” tau radiotracer [119]. However, Soleimani-Meigooni et al. [217] reported Flortaucipir binding in a patient carrying an S305I mutation associated with 4R tau pathology, suggesting that tau-PET could not clearly differentiate between FTLD-tau subtypes.

## Neuroimaging Heterogeneity Within FTD Clinical Phenotypes: Data-Driven Approaches

In parallel to the aforementioned studies looking at neuroimaging patterns in groups of patients based on their neuropathological subtype, another approach has emerged: using neuroimaging data itself to identify subgroups of patients. This approach has gained much interest in the Alzheimer’s field in recent years [241, 242], and some groups have applied similar data-driven methods to patients within specific FTD syndromes.

### bvFTD

In those with a clinical diagnosis of bvFTD, the patterns of neurodegeneration are quite heterogenerous. A 2009 study by Whitwell et al. [130] of 66 subjects diagnosed with bvFTD found four distinct anatomical subtypes based on the pattern of atrophy; all were associated with significant behavioral abnormalities, though memory, executive function, and language deficits were variable across subtypes. The frontal dominant subtype (*n* = 21) showed atrophy largely restricted to the frontal lobes, though the parietal lobes were also affected to a mild degree. The frontotemporal subtype (*n* = 12) also demonstrated a substantial degree of frontal atrophy and mild parietal atrophy, but also demonstrated significant atrophy of the temporal lobes, whereas the frontal dominant subtype did not. These two subtypes were associated with similar deficits in executive functioning, but the frontotemporal subtype had greater memory and language deficits compared to the frontal dominant subtype. The temporofrontoparietal subtype (*n* = 27), as the name suggests, showed frontoparietal atrophy with additional involvement of the medial temporal lobes, though frontal lobe atrophy was not as severe as that seen in the frontal dominant and frontotemporal subtypes. Executive functioning deficits were less severe in the temporofrontoparietal subtype compared to the frontal dominant and frontotemporal subtypes, and mamory and language deficits were similar to that seen in the frontotemporal subtype. The temporal dominant subtype (*n* = 6) showed atrophy that was entirely localized to the temporal lobes and much more pronounced than the other three subtypes. Memory and language deficits were most prominent in the temporal dominant subtype, whereas executive functioning deficits were mild.

A retrospective observational study by Ranasinghe et al. [243] classified 90 subjects who met criteria for bvFTD based on their patterns of gray matter volume using a principal component analysis using 18 regions of interest. Four subtypes were identified, with atrophy patterns mainly overlapping with the salience network (*n* = 21 with a predominant frontal/temporal pattern, *n* = 27 with a predominant frontal pattern), which corresponded to the frontal dominant and frontotemporal subtypes described by Whitwell et al. Other patients had atrophy located in the semantic appraisal network (*n* = 8), comparable to Whitwell’s temporal dominant subtype or predominant subcortical atrophy (*n* = 30). In general, the frontotemporal and temporal subtypes had the greatest frequency of most core diagnostic features of bvFTD, though executive dysfunction was much more prominent in the frontal and subcortical subtypes. A subset of 24 patients had available neuropathological data, with limited statistical power to detect differences. Yet, it should be noted that each of the four subtypes included both patients with FTLD-tau and FTLD-TDP43, suggesting that these imaging-based clusters were not simply reflective of neuropathological subtypes.

In 2016, Cerami et al. [244] conducted a retrospective study of 52 subjects who fulfilled Rascovsky [4] criteria for probable bvFTD, demonstrating that clinical phenotypes are correlated with specific FDG-PET patterns at the individual level. In this study, two distinct subtypes were found based on FDG-PET, a frontal (*n* = 25) and temporolimbic (*n* = 27) subtype. Both subtypes were associated with hypometabolism of the insula, anterior cingulate cortex, ventromedial PFC, nucleus accumbens, and thalamus. However, the frontal subtype demonstrated more pronounced hypometabolism of the PFC, whereas the temporolimbic subtype was predominantly demonstrated hypometabolism of the medial temporal lobe. The neuropsychological profiles of these two subtypes showed similar degrees of impaired empathy/sympathy, socioemotional deficits, and disinhibition and apathy, though isolated apathy was more prevalent in the frontal subtype and isolated disinhibition was present only in the temporolimbic subtype. Executive dysfunction and immediate recall were significantly more impaired in the frontal subtype, whereas delayed recall was more impaired in the temporal subtype. It is important to note that these subtypes have not been studied in pathology-confirmed cases, so it is unclear whether the differences seen are related to different neuropathological subtypes, or another causes of variability.

### svPPA

In cases of svPPA, atrophy is predominantly asymmetric and affects the anterior temporal lobe of the language-dominant cerebral hemisphere (generally the left hemisphere) in most cases. The neuropathological process causing this syndrome affects the non-language dominant cerebral hemisphere in about 40% of cases [132, 245]. When the non-language dominant anterior temporal lobe is affected, patients’ early presentation differs from svPPA. Rather than presenting as language impairment, early symptoms typically include impairment of facial recognition, loss of semantic knowledge regarding specific people, and behavioral changes similar to those seen in bvFTD [132, 181, 245, 246]. Regardless of which hemisphere is primarily affected, symptoms increasingly overlap as the disease progressively involves the contralateral hemisphere [247].

### nfvPPA

In cases of nfvPPA, structural and metabolic imaging reveals a left-predominant decline in the frontal lobe involving the pars opercularis, insula, middle frontal gyrus, and supplementary motor area [86]. In a 2019 study by Matias-Guiu et al. [248], it was found that in those with nfvPPA, two subtypes could be differentiated based on clustering of FDG-PET hypometabolism. In the first cluster, involvement of the left frontal lobe was predominant, impacting the left superior and inferior frontal gyri, insula, anterior cingulate, left caudate, and the left middle and medial frontal gyri. The second cluster revealed hypometabolism in the left superior, middle, medial, and inferior frontal gyri as well, but also in the left precentral and right superior frontal gyri. Interestingly, while both subtypes were associated with agrammatic or effortful, halting speech, only the second subtype was associated with apraxia of speech.

## Summary and Perspectives

Frontotemporal dementia is an umbrella term for several clinical syndromes, including bvFTD, svPPA, and nfvPPA, that are clinically,pathologically, and radiologically heterogeneous. Neuroimaging plays a key role in evaluating patients with FTD and can help clarify the clinical syndrome and underlying pathology, particularly in cases with distinct patterns of atrophy strongly associated with a specific pathology. However, it should be noted that the patterns of neurodegeneration are indirect markers of pathologic subtypes and are not necessarily diagnostic for a specific clinical syndrome. For example, FTLD-TDP type A is commonly associated with both bvFTD and nfvPPA. As such, the clinical manifestations must be considered in addition to the neuroimaging findings. In addition, the current literature on the association between imaging and neuropathology is based on small patient samples, with most groups being smaller than 20 patients, or even smaller than 5 patients when comparing neuropathological subtypes [249]. Consequently, most published studies had limited statistical power to detect group differences: when comparing two groups of 15 patients each using a two-tailed two sample *t*-test, one can expect to detect effect sizes ≥ 1.06 (with a power of 80% and α = 0.05). It should therefore be acknowledged that only large effect sizes have been reliably identified and that the absence of a statistically significant difference in a given measure or brain region should be interpreted with caution, recognizing the likelihood of false negative findings.

In FTLD-TDP, the three subtypes responsible for the majority of cases (A, B, and C) show group-level differences in patterns of neurodegeration. Type A is associated with asymmetric degeneration of the frontotemporal lobes as well as involvement of the parietal lobes and manifests most often as bvFTD or nfvPPA. Type B is associated with relatively symmetric medial and lateral frontal lobe atrophy as well as significant involvement of subcortical structures and manifests most often as bvFTD. Type C is associated with severe, asymmetric atrophy of the left or right anterior temporal lobes with a strong anterior–posterior gradient. Type C manifests as svPPA if the language dominant hemisphere is predominantly affected, or as impairment of facial recognition, loss of semantic knowledge regarding specific people, and behavioral changes similar to those seen in bvFTD if the non-language dominant hemisphere is predominantly affected.

In FTLD-tau, Pick’s disease, PSP, and CBD are responsible for the majority of cases. Pick’s disease (3R tau) is associated with a striking degree of frontotemporal atrophy generally beyond that seen in the other FTLD variants and manifests most commonly as bvFTD though nfvPPA, svPPA, and CBS also occur. PSP-bvFTD is most commonly associated with atrophy of the posterior cerebellum with relatively mild frontal lobe atrophy and sparing of the temporal lobes. CBD-bvFTD is associated with widespread atrophy symmetrically involving the prefrontal cortex, peri-Rolandic cortex, and striatum. In PSP-nfvPPA and CBD-nfvPPA, atrophy predominantly affects the left interior frontal gyrus, though atrophy in CBD-nfvPPA is distributed more widely.

In cases of aFTLD-U, there is extensive atrophy of the bilateral frontal and temporal lobes and caudate. Caudate atrophy is typically more severe compared to FTLD-TDP and FTLD-tau variants. Patients commonly develop symptoms before 40 years of age and typically meet clinical criteria for bvFTD.

In spite of these group-level patterns, there is an emerging focus on relatively unexplored factors driving heterogeneity within clinically or pathologically defined groups, or in the association between neuroimaging and clinical symptoms. A recent study by Illán-Gala et al. [250] found that females had a greater degree of atrophy compared to males while having similar clinical characteristics, suggesting that females have a greater degree of cognitive reserve. Another recent study by Lee et al. [251] explored the differences in neuroimaging features between early- and late-onset nfvPPA. The authors found that those with early-onset nfvPPA had a greater reduction of cortical thickness of the left perisylvian, lateral and medial prefrontal, temporal, posterior cingulate, and precuneus regions, despite having the same degree of clinical impairment. This observation might be due to differences in neuropathological subtype between early and late-onset nfvPPA or by a higher cognitive resilience in younger patients.

Despite the recent focus on these previously unexplored factors, there are many areas in which further studies are needed. One area that is significantly understudied is the impact of linguistic, geographic, social, and ethnoracial factors on neurodegenerative diseases, particularly regarding their neuroimaging signature. This is due to the fact that most of the neuroimaging research is being conducted in western countries, where study samples are not reflective of the overall population with an over-representation of white and educated individuals that biases neuroimaging findings [252]. As such, few studies have explored the associations between ethnoracial factors and structural or functional imaging, leaving a glaring gap in our knowledge of how these diseases might affect different groups [253, 254]. Lastly, the development of data-driven methods to directly identify subgroups of patients within a given clinically or even neuropathologically defined subtype [241, 255, 256] together with the constitution of large multicenter cohorts and data sharing opportunities [257] will help us better characterize the heterogeneity of FTLD pathophysiological processes.

## Supplementary Information

Below is the link to the electronic supplementary material.

Supplementary file1 (PDF 1225 KB)

Supplementary file2 (PDF 1225 KB)

Supplementary file3 (PDF 1225 KB)

Supplementary file4 (PDF 1225 KB)
